# Ultrasound-guided stellate ganglion block for refractory sympathetically mediated arrhythmia following acute myocardial infarction: a tutorial and examples

**DOI:** 10.3389/fcvm.2026.1846647

**Published:** 2026-06-19

**Authors:** Ranwei Yao, Yingjie Che, Xiuling Wu

**Affiliations:** Department of Anesthesiology and Perioperative Medicine, Shihezi People’s Hospital, Shihezi, China

**Keywords:** acute myocardial infarction, refractory ventricular arrhythmia, stellate ganglion block, sympathetic arrhythmia, sympathetic storm, ultrasound guidance

## Abstract

**Introduction:**

Sympathetic arrhythmia represents a life-threatening complication following acute myocardial infarction (AMI), which is characterized by recurrent episodes of electrical dysrhythmias that are often refractory to antiarrhythmic medications and thereby necessitate repeated electrical cardioversion or defibrillation. This case series aims to evaluate the efficacy and safety of ultrasound-guided stellate ganglion block (SGB) as a rescue therapy for refractory ventricular arrhythmias and one case of rapid atrial flutter following AMI.

**Methods:**

We retrospectively analyzed five AMI patients with refractory sympathetically arrhythmia who failed standard medical management. All patients were of Han ethnicity from China, consisting of three males and two females, with ages ranging from 29 to 87 years. All patients underwent bedside ultrasound-guided SGB (unilateral or bilateral) with 1% lidocaine hydrochloride. The primary outcomes were immediate termination of sympathetically arrhythmia and 24-hour arrhythmia-free survival.

**Results:**

SGB successfully aborted electrical storm in all 5 patients (100%). The median number of defibrillations per patient decreased significantly from 4 [interquartile range (IQR): 2.5–21] before SGB to 0 (IQR: 0–1) after SGB. The development of ipsilateral Horner syndrome in all cases confirmed successful sympathetic blockade, and no procedure-related complications were observed.

**Discussion:**

SGB is a safe and effective bedside intervention for terminating and stabilizing refractory sympathetically arrhythmia in post-AMI patients. It can serve as an important adjunctive therapy to reduce the need for repeated electrical defibrillation. Further prospective randomized controlled studies are warranted to validate its clinical application in acute arrhythmia management.

## 1 Introduction

Sympathetic arrhythmias encompass a variety of electrical abnormalities, including sinus tachycardia, atrial or ventricular premature beats, atrial tachycardia, supraventricular tachycardia, atrial fibrillation, ventricular tachycardia, sympathetic electrical storm, and catecholaminergic polymorphic ventricular tachycardia, among which sympathetic electrical storm is considered the most lethal subtype. Sympathetic storm (SS) which is marked by a series of arrhythmias in the ventricles and is commonly characterized by three or more arrhythmias in 24 h. It is a severe form of cardiac electrical instability that most often occurs in acute myocardial infarction (AMI) (1, 2) situation. Despite conventional advanced cardiac life support, such as antiarrhythmic medications and repeated shock, SS often does not respond and has high mortality rates (3). Stellate ganglion block (SGB), a procedure that modulates cardiac sympathetic tone, has renewed promise as a method of refractory arrhythmia intervention (4, 5). With the introduction of ultrasound guidance, the safety and accuracy of this block have greatly enhanced, as it allows visualization on a real-time basis on the location and dissemination of the local anesthesia and positioning of the needle, thus reducing risks. Its efficacy in treating electrical storms has only recently been supported by clinical studies such as the STAR trial (6). Nevertheless, there is a dearth of reports on its use as an AMI-induced SS treatment at the acute stage, especially as a bedside rescue treatment. Furthermore, emerging evidence suggests that SGB may also be effective in managing supraventricular arrhythmias, such as atrial fibrillation and atrial flutter, by modulating cardiac sympathetic activity at a broader level (7, 8). This case series seeks to fill this gap by providing our clinical experience with five AMI patients. Proving the critical nature of ultrasound-guided SGB in terms of stabilizing the highly vulnerable population in a very short amount of time when other conventional therapies in use prove unsuccessful (9). While sympathetic storm is classically defined by recurrent ventricular arrhythmias, we also included one patient with rapid atrial flutter (Case 5) to explore the potential broader application of SGB for post-AMI tachyarrhythmias.

## 2 Case presentation

### 2.1 SGB procedure details

All ultrasound-guided SGB procedures were performed at the bedside or in the catheterization laboratory by experienced anesthesiologists trained in this technique. With the patient in a supine position and the neck slightly rotated to the contralateral side, the neck skin was routinely disinfected. A high-frequency linear array ultrasound probe (Mindray M9) was used (Figure 1A). The probe, covered with a sterile sheath and coupling gel, was placed transversely at the level of the cricoid cartilage, corresponding to the sixth cervical vertebra(C6), to identify the transverse process of C6, the C6 nerve root, and the carotid artery (Figure 1B). The artery was positioned in the center of the screen. The needle insertion point was determined lateral to the probe (Figures 1C–E). Under real-time ultrasound guidance, a 27-gauge needle was advanced using an in-plane technique from the lateral edge of the probe towards the prevertebral fascia overlying the longus colli muscle. After confirming negative aspiration for blood, 5 mL of 1%lidocaine hydrochloride (Batch No. D32507092, Hubei Jinyao Pharmaceutical Co., Ltd.) was injected. Regarding the rationale for the chosen dose, we selected 5 mL of 1% lidocaine (50 mg) based on our clinical experience and institutional practice, which has demonstrated effective sympathetic blockade with a lower volume to minimize the risk of local anesthetic toxicity, especially in hemodynamically unstable post-AMI patients. While the STAR trial and other studies have used higher doses or combinations with other anesthetics, our approach prioritizes safety in the acute setting, and the consistent achievement of Horner syndrome and arrhythmia termination in all cases confirms the adequacy of this dose. Successful spread of the local anesthetic was confirmed ultrasonographically by visualizing a crescent-shaped or fusiform hypoechoic fluid collection between the prevertebral fascia and the longus colli muscle, posterior to the carotid sheath, often spreading craniocaudally between the C6 and C7 vertebral levels (Figures 1E–K). The immediate development of ipsilateral Horner syndrome (ptosis, miosis, and anhidrosis) was considered clinical confirmation of a successful block.

**Figure 1:**
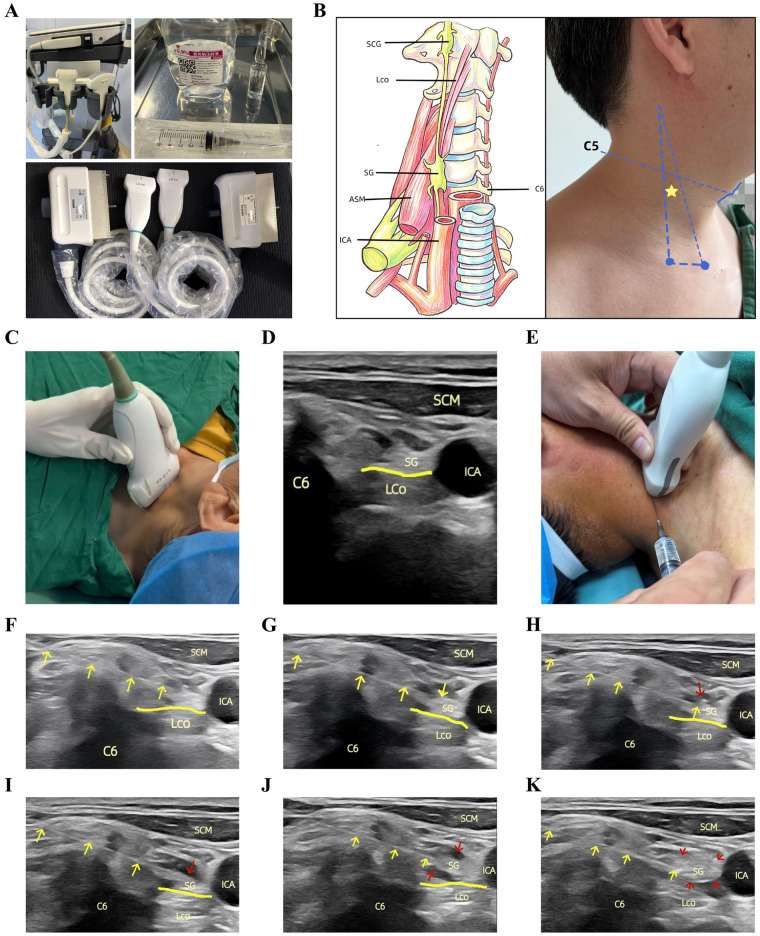
Step-by-step approach for ultrasound-guided stellate ganglion block in refractory sympathetically mediated arrhythmia following acute myocardial infarction. **(A)** Preparation of equipment and drugs (including ultrasound machine with high-frequency linear probe, sterile sheath, coupling gel, 27-gauge needle, 1% lidocaine hydrochloride); **(B)** anatomy and surface localization. The blue line marks the thyroid cartilage prominence (C6 level). The dotted area indicates the sternocleidomastoid muscle (SCM). The yellow asterisk area marks the approximate superficial projection of the stellate ganglion (SG). Relevant deep structures are labeled: Lco: Longus colli muscle, SCG: Superior Cervical Ganglion, ASM: Anterior scalene muscle; **(C)** placement of the ultrasound probe on the body surface. The ultrasound probe is placed transversely over the posterior border of the SCM at the level of the cricoid cartilage (C6); **(D)** ultrasound schematic diagram. The yellow line indicates the prevertebral fascia (PVF). Key structures visualized include the internal carotid artery (ICA), the C6 transverse process, and the longus colli muscle (Lco); **(E)** entry site and needle insertion technique. An in-plane approach is used, with the needle inserted from the lateral edge of the probe; **(F–K)** ultrasound-guided SGB schematic (long-axis view). Yellow arrows: needle tract; Yellow line: prevertebral fascia; Red arrows: injectate diffusion. **(F)** Needle tract is visualized advancing toward the target. **(G)** The needle tip approaches the stellate ganglion located within the prevertebral fascia overlying the longus colli muscle. **(H)** Partial injectate (1% lidocaine) is deposited around the stellate ganglion. **(I,J)** The injectate gradually spreads to surround the stellate ganglion. **(K)** Full circumferential coverage of the stellate ganglion by the injectate is achieved, confirming successful blockade. ICA, internal carotid artery; C6, sixth cervical vertebra; Lco, longus colli muscle; PVF, prevertebral fascia; SCM, sternocleidomastoid muscle; SG, stellate ganglion.

### 2.2 Case 1

A 29-year-old female patient was selected, who was diagnosed with “Acute ST Elevation Myocardial Infarction (Anterior Wall) Killip IV”, having a history of “Type 2 Diabetes, Obesity, and Non-severe Community-Acquired Pneumonia” A dominant right coronary artery was identified with 90% stenosis in the left anterior descending artery (LAD) during emergency percutaneous coronary intervention (PCI). The vessel was successfully opened after 101 min. However, 30 min after reperfusion, the patient experienced a sympathetic storm, necessitating 21 episodes of electrical defibrillation. A bilateral stellate ganglion block (SGB)was administered by an anesthesiologist (5 mL of 1% lidocaine on the left side in the catheterization laboratory, followed by 5 mL of 1% lidocaine on the right side after 20 min), and endotracheal intubation was performed for 24 h to successfully terminate ventricular fibrillation (Figure 2).

**Figure 2:**
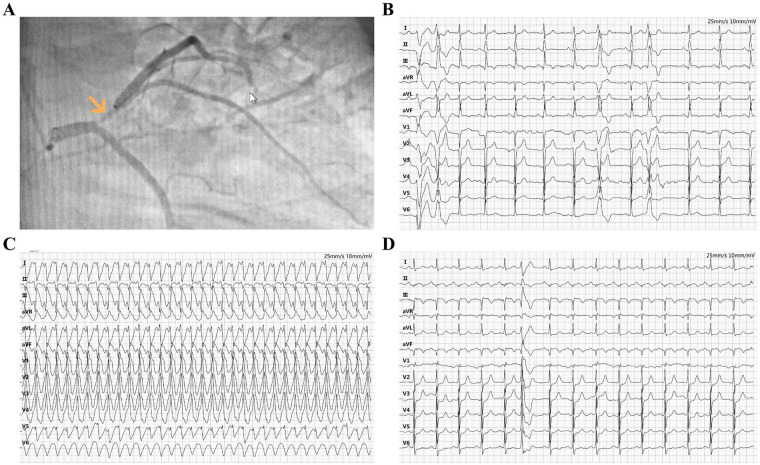
Case 1. **(A)** Lesion location shown on coronary angiography (orange arrows). **(B)** Acute anterior myocardial infarction with T-wave changes at admission. **(C)** The patient's electrocardiogram before treatment: ventricular tachycardia with a heart rate of 192 beats per minute during percutaneous coronary intervention (PCI). **(D)** Significantly decreased heart rate 10 min after ultrasound-guided stellate ganglion block.

### 2.3 Case 2

A 47-year-old male patient was selected, who was diagnosed with “Acute ST Elevation Myocardial Infarction (Inferior Wall) Killip IV”, having a history of “Grade 3 Hypertension (Very High Risk) and Pulmonary Infection”. A dominant right coronary artery was identified with plaque in the mid-LAD during emergency PCI, 90% stenosis in the proximal left circumflex artery (LCX), 100% occlusion in the obtuse marginal (OM) branch, and 60% stenosis with severe tortuosity in the mid-right coronary artery (RCA) and distal occlusion. The vessel was successfully opened after 185 min. However, 60 min after reperfusion, the patient experienced a sympathetic storm. The left SGB was administered by an anesthesiologist (5 mL of 1% lidocaine) in the catheterization laboratory, and an implantable cardioverter defibrillator (ICD) was implanted after 4 episodes of electrical defibrillation. Five hours later, the patient experienced a sympathetic storm. After one episode of cardiopulmonary resuscitation and three episodes of electrical defibrillation, bedside SGB was performed by the anesthesiologist, administering left SGB (5 mL of 1% lidocaine), and ventricular fibrillation and tachycardia were successfully terminated for 24 h (Figure 3).

**Figure 3:**
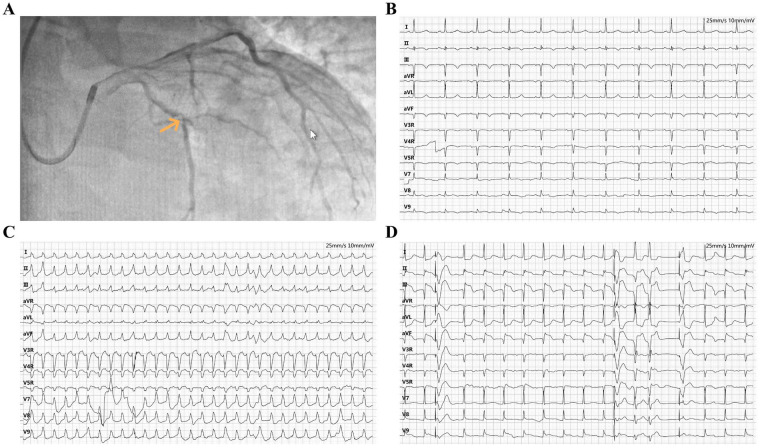
Case 2. **(A)** Lesion location shown on coronary angiography (orange arrows). **(B)** Initial ECG showing acute inferior myocardial infarction with ST-segment changes. **(C)** The patient's electrocardiogram before treatment: ECG revealed paroxysmal supraventricular tachycardia with a heart rate of 180 bpm. **(D)**. Significantly decreased heart rate 10 min after ultrasound-guided stellate ganglion block [acute inferior myocardial infarction, frequent ventricular premature beats (some in couplets), heart rate 104 bpm].

### 2.4 Case 3

A 54-year-old male patient was diagnosed with “Acute ST Elevation myocardial infarction (High Lateral Wall) Killip I”, with no underlying diseases. In emergency PCI, the coronary circulation was of left-dominant type. The left main coronary artery (LMCA) demonstrates 70% stenosis with plaque rupture at its body segment. The proximal left anterior descending artery (LAD) has 80% luminal stenosis, and the intermediate branch is totally occluded (100% stenosis). The proximal and distal segments of the left circumflex artery (LCX) exhibit 80% and 90% stenosis, respectively. The mid-right coronary artery (RCA) shows diffuse 90% stenosis. The vessel was successfully opened after 90 min. However, 45 min after reperfusion, the patient experienced Paroxysmal supraventricular tachycardia. In the coronary care unit (CCU), bilateral SGB was administered by an anesthesiologist (4 mL of 1%lidocaine on the left side, followed by 4 mL of 1%lidocaine on the right side after 20 min) and endotracheal intubation was performed, successfully terminating ventricular tachycardia for 24 h (Figure 4).

**Figure 4:**
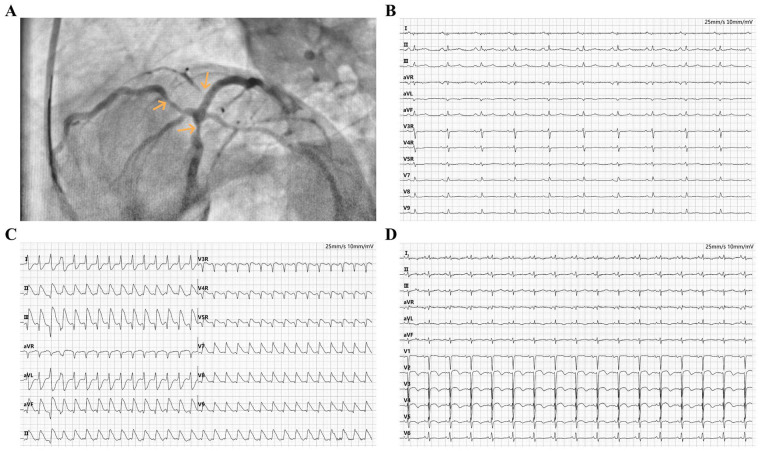
Case 3. **(A)** Lesion location shown on coronary angiography (orange arrows). **(B)** ECG: acute high lateral myocardial infarction with T-wave changes. **(C)** The patient's electrocardiogram before treatment: paroxysmal supraventricular tachycardia (PSVT) with ST-T changes, HR: 179 bpm. **(D)**. Significantly decreased heart rate 10 min after ultrasound-guided stellate ganglion block: HR: 99 bpm with ST-T changes.

### 2.5 Case 4

A 77-year-old male patient was diagnosed with “Acute ST Elevation Myocardial Infarction (Inferior Wall) Killip III”, with a history of Mild Mitral Regurgitation and Non-severe Community-Acquired Pneumonia. The patient experienced ventricular tachycardia, and electrical defibrillation was performed three times after admission. In the CCU, bilateral SGB was administered by an anesthesiologist (5 mL of 1%lidocaine on the left side, followed by 5 mL of 1% lidocaine on the right side after 15 min), and endotracheal intubation was performed, successfully terminating ventricular tachycardia for 24 h. Three days later, PCI revealed a dominant right coronary artery with 90% stenosis in the LCX and 90% proximal stenosis in the RCA, with the vessel successfully opened after 103 min (Figure 5).

**Figure 5:**
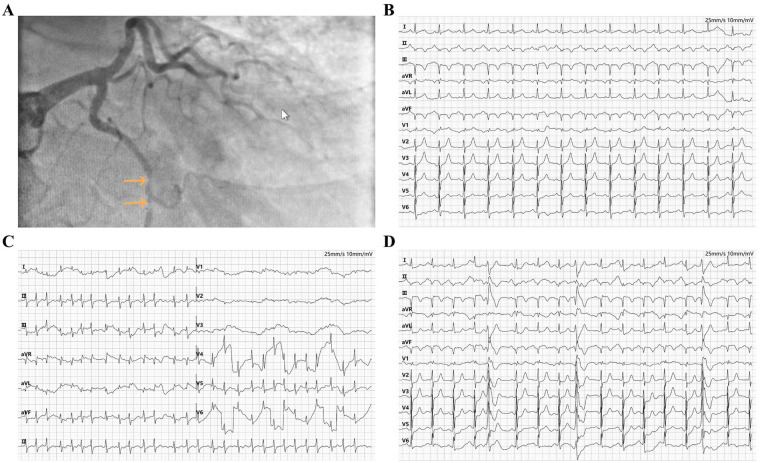
Case 4. **(A)** Lesion location shown on coronary angiography (orange arrows). **(B)** ECG: acute inferior myocardial infarction. **(C)** The patient's electrocardiogram before treatment: sinus rhythm plus ectopic rhythm; sinus tachycardia, frequent atrial premature contractions, paroxysmal atrial tachycardia, ST-segment changes, HR: 176 bpm. **(D)** Significantly decreased heart rate 10 min after ultrasound-guided stellate ganglion block: HR: 100 bpm with ST-T changes.

### 2.6 Case 5

An 87-year-old female patient was selected, who was diagnosed with “Acute ST Elevation Myocardial Infarction (Extensive Anterior + True Posterior + High Lateral Wall) Killip IV”, having a history of “Pulmonary Embolism, Type 2 Diabetes, and Moderate Malnutrition”. PCI was not performed because of the patient's advanced age. The patient experienced rapid atrial flutter and electrical defibrillation was performed once after admission. Given the presence of a right internal jugular venous catheter, ultrasound-guided stellate ganglion block could not be performed on the right side; therefore, only left-sided stellate ganglion block was administered in this patient.

In the CCU, left SGB was administered by an anesthesiologist (4 mL of 1% lidocaine), successfully terminating the rapid atrial flutter. The patient experienced rapid atrial flutter again forty-eight hours later and underwent electrical cardioversion twice. Left SGB was administered by an anesthesiologist in the CCU (4 mL of 1% lidocaine), successfully terminating rapid atrial flutter. The patient experienced rapid atrial flutter again four days later and was administered a left SGB (4 mL of 1% lidocaine). The patient did not experience recurrence until discharge (Figure 6).

**Figure 6:**
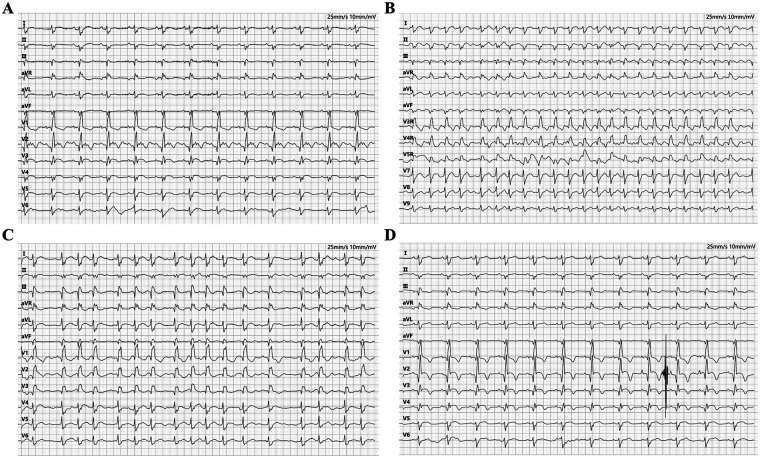
Case 5. **(A)** The patient was diagnosed with acute extensive anterior wall myocardial infarction at admission. **(B,C)**. The patient's electrocardiogram before treatment: rapid atrial flutter and short-run atrial tachycardia, heart rate: 136 bpm. **(D)** Significantly decreased heart rate 10 min after ultrasound-guided stellate ganglion block: ECG: acute high lateral and extensive anterior myocardial infarction, HR: 83 bpm.

## 3 Results

Five patients with AMI-refractory Sympathetically mediated arrhythmia were included in this study. Table 1 summarizes the baseline features, the myocardial infarction features, and initial clinical presentations of all patients. Recurrent life-threatening arrhythmias developed in all the patients despite normal pharmacological treatment and electrical cardioversion. Subsequently, they were subjected to bedside ultrasound-guided stellate ganglion block (SGB) as rescue therapy. Table 2 provides sufficient information regarding the procedures performed and clinical outcomes. Among the four patients with ventricular arrhythmias (Cases 1–4), SGB successfully aborted the electrical storm in all cases (100%). For Case 5 (rapid atrial flutter), SGB effectively terminated the arrhythmia after each of the three episodes. The objective confirmation of a successful blockade was the immediate development of ipsilateral Horner syndrome in all patients, and the median onset time was less than 3 min. The need for electrical defibrillation after SGB decreased dramatically. There was a reduction in the median number of consecutive episodes of defibrillation/storm to 4 [IQR:2.5–21] pre-SGB and 0 [IQR:0–1] post-SGB. The results of repeated measures analysis of variance in this study showed that the cardiac structure and function indexes, including LVEF, LVEDD, LVESD and WMSI, were significantly improved from 24 h after surgery to 30-day follow-up compared with the preoperative baseline level (*P* < 0.05), while there was no statistical difference in the above indexes immediately after surgery (*P* > 0.05) (Table 3).

**Table 1:** Patient baseline characteristics, myocardial infarction details and clinical presentation.

Patient No.	1	2	3	4	5
Age (years)/Sex	29/F	47/M	54/M	77/M	87/F
Comorbidities	T2DM, Obesity, Community-acquired pneumonia	Grade 3 Hypertension, Pulmonary infection	None	Mild mitral regurgitation, Community-acquired pneumonia	T2DM, PE, DVT, Osteoporosis, Moderate malnutrition
Infarct Location (Killip Class)	Anterior wall (IV)	Inferior wall (IV)	High lateral wall (I)	Inferior wall (III)	Extensive anterior + true posterior + high lateral wall (IV)
LVEF (%)	27	45	55	40	51
Wall Motion Abnormalities	Anterior wall akinesia	Inferior wall akinesia	No obvious abnormality	Global hypokinesia	Anterior & inferior wall akinesia
Culprit Artery	LAD	RCA	LAD/LCX/RCA	LCX/RCA	Not performed
Symptom to Reperfusion Time (min)	120	240	90	103	N/A
SS Onset after Reperfusion (min)	30	60	45	N/A	N/A
Initial Arrhythmia	Ventricular fibrillation	Ventricular fibrillation	Ventricular tachycardia	Ventricular tachycardia	Rapid atrial flutter
Patient No.	1	2	3	4	5
Age (years)/Sex	29/F	47/M	54/M	77/M	87/F
Comorbidities	T2DM, Obesity, Community-acquired pneumonia	Grade 3 Hypertension, Pulmonary infection	None	Mild mitral regurgitation, Community-acquired pneumonia	T2DM, PE, DVT, Osteoporosis, Moderate malnutrition

**Table 2:** Treatment details and clinical outcomes.

Patient No.	1	2	3	4	5
Pre-SGB AADs (Drug, Dose)	Amiodarone IV 300 mg	Amiodarone IV 300 mg	Amiodarone IV 300 mg	Metoprolol PO 25 mg	Amiodarone IV 150 mg
Pre-SGB Vasopressors (Drug, Dose)	Norepinephrine 0.1 μg/kg/min	Norepinephrine 0.08 μg/kg/min	None	Dopamine 5 μg/kg/min	None
Pre-SGB Defibrillation/Cardioversion Count	21	4 (1st episode) + 3 (2nd episode) = 7	0 (VT without defibrillation)	3	1 (1st episode) + 2 (2nd episode) = 3
SGB Procedure (Side, Lidocaine Dose)	Bilateral, 5 mL each	Left, 5 mL (1st); Left, 5 mL (2nd)	Bilateral, 4 mL each	Bilateral, 5 mL each	Left, 4 mL (1st); Left, 4 mL (2nd); Left, 4 mL (3rd)
Horner's Syndrome (Y/N, Onset Time)	Y, <2 min (bilateral)	Y, <2 min	Y, 1 min (bilateral)	Y, <2 min (bilateral)	Y, 1 min
SS Terminated (Y/N)	Y (1st block)	N (1st block); Y (2nd block)	Y (1st block)	Y (1st block)	Y (1st/2nd/3rd block)
Post-SGB Defibrillation/Cardioversion Count	0	0 (after 2nd block)	0	0	0 (after each block)
Subsequent Recurrence Intervals (h)	No recurrence	5 (after 1st block); No recurrence (after 2nd block)	No recurrence	No recurrence	48 (after 1st); 48 (after 2nd); No recurrence (after 3rd)
Additional SGB (s)	0	1	0	0	2
Procedure-Related Complications	None	None	None	None	None
Final Outcome/Disposition	Discharged	ICD implanted, discharged	Discharged	Discharged	Discharged
Pre-SGB AADs (Drug, Dose)	Amiodarone IV 300 mg	Amiodarone IV 300 mg	Amiodarone IV 300 mg	Metoprolol PO 25 mg	Amiodarone IV 150 mg
Pre-SGB Vasopressors (Drug, Dose)	Norepinephrine 0.1 μg/kg/min	Norepinephrine 0.08 μg/kg/min	None	Dopamine 5 μg/kg/min	None

Case 5 is presented separately as it involves atrial flutter rather than ventricular arrhythmia.

**Table 3:** Perioperative measurement results of left ventricular function parameters in surgical patients at preoperative baseline and different postoperative time points (mean ± SD).

Index (Abbreviation)	Unit	Preoperative (Baseline)	Immediately Postoperative	24 H Postoperative	30-day follow-up postoperative
Left Ventricular Ejection Fraction (LVEF)	%	41.2 ± 7.6	42.6 ± 8.5*	51.8 ± 9.3*	53.6 ± 11.7*
Left Ventricular End-Diastolic Diameter (LVEDD)	mm	59.0 ± 9.0	59.0 ± 9.0	55.0 ± 8.0*	53.2 ± 9.2*
Left Ventricular End-Systolic Diameter (LVESD)	mm	49.1 ± 9.7	48.7 ± 10.2	42.2 ± 9.2*	40.3 ± 10.9*
Wall Motion Score Index (WMSI)	Point	1.8 ± 0.5	1.7 ± 0.5*	1.3 ± 0.3*	1.3 ± 0.3*

* *P* < 0.05 vs. Preoperative (Baseline) group. The overall difference between groups was tested by repeated measures analysis of variance, and the pairwise comparison was performed by LSD-t test.

Regarding the duration of efficacy, the arrhythmia-free period following the first SGB procedure for each patient is detailed in Table 2. Patients 2 and 5 experienced recurrent arrhythmia after 5 and 48 h, respectively. These recurrences were effectively treated with follow-up SGBs, and no additional episodes were reported until discharge. Unilateral (left-sided) and bilateral SGB techniques were both useful in the acute stabilization of arrhythmia. None of the patients experienced any complications associated with the procedure, including vascular puncture, hematoma, or respiratory distress.

After discharge, all patients were followed up for a period of 3 months through outpatient visits or telephone interviews. No recurrence of life-threatening arrhythmias or late complications related to the SGB procedure was reported during this follow-up period. The absence of life-threatening arrhythmia recurrence during the 3-month follow-up period is interpreted as a composite outcome, reflecting successful acute termination of the storm by SGB followed by the establishment of effective long-term management, including optimized pharmacotherapy, complete revascularization where applicable, and device therapy (ICD) when indicated.

It is important to note that while SGB was temporally associated with the termination of the sympathetic storm and a dramatic reduction in defibrillation requirements, patients received multiple concomitant therapies (e.g., antiarrhythmic drugs, vasopressors, mechanical ventilation, PCI). Therefore, this case series demonstrates a strong temporal association rather than establishing a sole causal effect of SGB.

## 4 Discussion

This case series indicates that ultrasound-guided stellate ganglion block (SGB) is a very powerful and safe rescue treatment for refractory sympathetic storm (SS) after AMI (10), especially when another pharmacotherapy and electrical cardioversion have been unsuccessful (11, 12). While the role of SGB in managing electrical storm is gaining acknowledgment (13), our case series provides specific insights into its application during the acute, vulnerable phase of AMI. The novelty of this report lies in detailing the feasibility and efficacy of bedside, ultrasound-guided SGB as a rescue therapy for AMI-induced refractory sympathetically arrhythmia, a context characterized by dynamic ischemia, reperfusion injury, and critical illness. This approach serves as a crucial stabilizing bridge in the intensive care setting, either until definitive revascularization can be achieved or as an adjunct prior to device implantation (e.g., ICD). Furthermore, the successful termination of recurrent atrial flutter in one patient (Case 5) extends the observed potential therapeutic scope of SGB beyond ventricular arrhythmias in the setting of acute cardiac ischemia, warranting further investigation.

Combined with the intervention regimen of this study and the whole clinical treatment course of patients, the long-term improvement of postoperative cardiac structure and function is more likely to be related to the continuous onset of standardized postoperative drug therapy, including anti-myocardial ischemia, antiarrhythmic, lipid-regulating and cardiac function-improving drugs, the effective control of underlying diseases, and the gradual recovery of patients' own myocardial function. The lidocaine stellate ganglion block used in this study is a short-acting and reversible nerve intervention, with limited duration of effect after a single block. No significant independent correlation between stellate ganglion block and long-term changes of postoperative cardiac structure was observed in this study (Table 4), and its core clinical benefits are still concentrated in the short-term effects such as rapid inhibition of sympathetic storm, stabilization of cardiac electrical activity, and reduction of the frequency of electrical defibrillation during the perioperative period.

**Table 4:** Statistical results of repeated measures ANOVA for left ventricular function parameters.

Index (Abbreviation)	*F* Value of repeated measures ANOVA	Overall *P* value	*P* value (vs. preoperative baseline) immediately postoperative	*P* Value (vs. preoperative baseline) 24 H postoperative	*P* Value (vs. preoperative baseline) 30-day follow-up postoperative
Left Ventricular Ejection Fraction (LVEF)	13.497	0	0.3509	0	0
Left Ventricular End-Diastolic Diameter (LVEDD)	3.3	0.0229	1	0.0158	0.0016
Left Ventricular End-Systolic Diameter (LVESD)	6.017	0.0008	0.8274	0.0004	0.0001
Wall Motion Score Index (WMSI)	12.206	0	0.2823	0	0

ANOVA, analysis of variance. The overall *P* value was derived from repeated measures ANOVA, and the pairwise comparison *P* values were derived from the *post-hoc* test between each postoperative time point and the preoperative baseline group.

Our results show a strong temporal association between SGB administration and the acute termination of sympathetically arrhythmia, accompanied by a significant decrease in defibrillation needs. However, given the retrospective nature of this series and the concurrent administration of other standard therapies (including antiarrhythmic medications, sedation/intubation, and revascularization), a definitive causal attribution to SGB alone cannot be made. The observed effect likely represents the contribution of SGB within a multifaceted treatment strategy. The main conclusion of our results is that SGB is a safe and effective bedside intervention for terminating and stabilizing refractory ventricular arrhythmias in post-AMI patients, with potential application for other post-AMI tachyarrhythmias that warrants further investigation. The physiological explanation is sufficiently established: heterogeneous cardiac sympathetic remodeling occurs as a result of myocardial infarction that preconditions regions of denervation and hyperinnervation that raise the electrophysiological heterogeneity and predisposition to malignant arrhythmias. SGB only partially inhibits the left stellate ganglion, the major sympathetic innervation to the ventricles, thereby decreasing sympathetic over governance and increasing ventricular fibrillation threshold. We have had experience consistent with an increasing body of evidence, for example, the STAR study by scientists who found the efficacy of multiple blocks compared to one injection is clearly demonstrated through our Cases 2 and 5 (14–17). The choice of unilateral (left-sided) vs. bilateral SGB was individualized based on operator preference, patient hemodynamic status, and safety considerations. Bilateral blockade was preferentially used in younger, hemodynamically stable patients with refractory ventricular arrhythmias to ensure maximal sympathetic suppression, while left-sided SGB was chosen in older or frail patients to reduce the risk of bilateral complications.

In addition, our research adds to the existing body of knowledge by indicating the possibility that bedside ultrasound-guided SGB can be used in a critical care environment as an essential step to the final treatment, such as ICD implantation. The effective treatment of a case with dominant atrial tachyarrhythmia (Case 5) also foreshadows the possible comparable extension of SGB to non-ventricular arrhythmia (possibly with a generalized low-gamma cardiac sympathetic activity) (18–20). This observation aligns with recent reports indicating that SGB can suppress supraventricular tachyarrhythmias, likely through generalized reduction of cardiac sympathetic outflow, thereby extending its potential therapeutic application beyond ventricular arrhythmias. We acknowledge that Case 5 (atrial flutter) represents a distinct arrhythmia type from the ventricular storm cases. The inclusion of this case was intended to explore the potential broader application of SGB for post-AMI tachyarrhythmias, as suggested by recent studies (18–20). However, we recognize that this reduces the internal consistency of the series. Therefore, we have presented the atrial flutter case separately in the analysis to avoid overgeneralization.

It is critical to interpret the long-term outcomes appropriately. The pharmacological effect of a lidocaine SGB is transient, typically lasting hours. Therefore, the reported freedom from life-threatening arrhythmias at the 3-month follow-up cannot be ascribed to the SGB procedure alone. Instead, it represents a sequential therapeutic success: the SGB served as a critical bridge therapy to acutely terminate the refractory electrical storm and stabilize the patient, creating a window of opportunity to implement or optimize definitive long-term management. In our series, this subsequent management included uptitration and sustained administration of antiarrhythmic drugs (e.g., amiodarone, beta-blockers), complete percutaneous coronary revascularization in four patients, and implantation of an ICD in one patient. The synergy between acute sympathetic modulation (SGB) and subsequent comprehensive medical, interventional, and device-based therapy likely underpinned the favorable long-term outcome, preventing the early recurrences often seen when SGB is used as a standalone intervention without addressing the underlying substrate.

This study has several limitations inherent to a retrospective case series, including a small sample size, the absence of a control group, and the concurrent use of multiple interventions (pharmacological therapy, PCI, intubation/sedation, ICD implantation). As such, while the temporal association between SGB and arrhythmia suppression is compelling, it does not prove causality. The resolution of arrhythmias may have been influenced by a combination of factors including reperfusion, hemodynamic stabilization, and sedation, in addition to the SGB. Prospective, randomized trials would also be justified in the future, as more definitive proof of the position of SGB in the AMI treatment paradigm would be available, and continuous blockade vs. intermittent boluses could be evaluated.

## Data Availability

The original contributions presented in the study are included in the article/Supplementary Material, further inquiries can be directed to the corresponding author.
